# A new secure authentication based distance bounding protocol

**DOI:** 10.7717/peerj-cs.517

**Published:** 2021-05-06

**Authors:** Ahmed Raheeq Sultan, Imran Rashid, Fawad Khan, Shahzaib Tahir, Maruf Pasha, Aiman Sultan

**Affiliations:** 1 Department of Information Security, National University of Sciences and Technology, Islamabad, Pakistan; 2 Department of Electrical Engineering, Institute of Space Technology, Islamabad, Pakistan; 3 Department of Information Technology, Bahauddin Zakariya University, Multan, Pakistan

**Keywords:** Information leakage, Cryptography, Position verification, Mafia Graud, Key exchange

## Abstract

Numerous systems are being employed in daily life where two entities authenticate each other over a range of distance. The distance involved is relatively small, but still attacks were documented. The distance bounding (DB) protocol was introduced to cater to security requirements. The schemes, however, are still prone to several threats; mainly the Relay Attack (Terrorist and Mafia Fraud). In Mafia Fraud, an attempts are made to get accepted as the prover either by replaying of messages or by the help a malicious key. In Terrorist fraud, an attempt is made to extract the secret from the verifying entity, either by extracting the key from the message captured or by physically tempering the verifying/proving entity. Therefore the mitigation of these attacks needs to be done; as to not put computational overhead on the scheme. The paper presents a comprehensive and comparative performance analysis of twelve DB protocols based on defined metrics. It also proposes a protocol which incorporates the design elements needed for added security, is computationally easy to implement and resistant to most of the threats mentioned. Analysis of the protocol is carried out against the security requirements.

## Introduction

With the advancement in technology, new innovations and ideas have been brought into the world. Where this has brought ease and comfort across the globe, it has also increased the chances of threats and theft effecting the overall security of the practice under consideration. Consider a scenario where two entities need to communicate over a distance. Prior to communication those entities need to authenticate each other, paving a way for trusted environment. In a real time networked environment, either wired or wireless, the provision of access control between two commodities is after the authentication and authorization phase. In daily life, there is often a need where one entity needs to verify another before giving access. For instance, the key-less entry system in today’s cars. The Electronic Car Unit (or abbreviated as ECU) would like to know that the person trying to gain access to the car is no more than a few meters away. For this, the ECU needs to determine a maximum limit of distance between the car and the driver (person carrying the key fob). For better understanding, take the example of an E-tag system used in vehicles. The system needs to be sure that the vehicle is near so that it may open the gate. Open the gate too early and there is a chance of malicious entry. Open the gate too late and the driver would have to wait. The first distance bounding protocol by Brands & Chaum (1993) addressed this issue. The protocol was first presented in 1993, and was thus a primitive approach of the problem but was lacking security and other constraint issues.

A basic distance bounding (DB) protocol consists of a Tag and a Reader where the two parties communicate over a range of distance. The whole process is based on exchange of challenges and received bits between both entities. The time of the journey is calculated; which form the basis of the protocol and enables the verifier to compute a maximum limit of distance between both parties. DB Protocols are cryptographic protocols that enable one party; the verifier “V” to verify a second party; the prover “P” (Capkun, El Defrawy & Tsudik, 2011), which is achieved by the help of the maximum limit applied on the distance between both. This works on the challenge bits sent and then received by the verifier after which the time taken by the entire journey is computed. The prover is then verified and given access. The process of the bit exchange is prone to security threats and different attacks can be launched on it.

There are several authentication protocols used in cryptographic systems, which aim to allow two entities to authenticate one another. There are many said protocols in practice which include the Point—to—Point Protocol PPP (Password Authentication Protocol PAP, Challenge Handshake Authentication Protocol CHAP, etc.) and Authentication, Authorization and Accounting AAA Protocol (RADIUS AND DIAMETER). These protocols are generally used in environments, where the question of resources is not an issue; because these work on challenge—response, based on symmetric keys (block ciphers). The real scenario, however, is quite different. The prover in this case, uses a simple device answering to any authentication request automatically.

The DB protocol under consideration, as described before, is a resource constrained protocol. Most of the authentication protocols cannot be implemented as such on the DB and therefore require modification at the very least. This is to be carried out in such a way that certain attacks which are possible on some of the authentication protocols can be mitigated. For example, the Man in the Middle Attack is possible on the PAP Protocol. This, therefore, concludes that the notion of simple authentication will not work and must be changed in compliance with the protocol’s controlled resource requirement. The authentication process as described before can be implemented with a message and some means to intermingle this message such that it is not easy for the attacker to use this for illicit purposes. The process of intermingling of this message; that will be communicated between the prover and the verifier, is what the security of the protocol is all about and forms the basis of our work. Table 1 enlists the different acronyms, abbreviations and explanations used in the entire paper.

**Table 1:** Table of acronyms/abbreviations.

Explainations	Acronyms/ abbreviations	Explainations	Acronyms/ abbreviations
Waters and Felten	WF	Decision Tress	DT
Čapkun and Hubaux	CH	Swiss Knife	SK
Avoine and Tchamkerten	ATP	Initialization Phase	IP
Brands and Chaum	BC	Rapid Bit Exchange Phase	RBEP
Hancke and Kuhn	HK	Authentication Phase	AP
Bussard and Bagga	BB	Mutual Authentication Phase	MAP
Nikov and Vauclair	NV	Mafia Fraud	MF
Munilla and Peinado	MP	Terrorist Fraud	TF
Singelée and Preneel	SP	Relay Attack	RA
Tu and Piramathu	TP	Denial of Service Attack	DOS
Length of bits	b	Distance Fraud	DF
Length of Nonce	N	Message Authentication Code	MAC
Location Manager	X.509	Public Key Infrastructure	PKI
Security Parameter	“n” and “k”	Basic Public Key Infrastructure	BPKI
Shared Secret	“s”	One Way Collision Resistant Hash	OWCRHF
Shared Key	“x” and “m”	Zero Knowledge Protocol	ZKP
Seed	“R” of length “k”	Symmetric Encryption	SE
Error Correction Code	(n,k)	Pseudo Random Functions	PRF
Long Term Key	L	Hash Message Authentication Code	HMAC
Hamming Distance	HD	Hash Function	H
Signatures	S	Error Correction Code	ECC
Generate Session Key	GSK	Node Capture Attack	NCA
Pre computed	PC	Without Mutual Authentication	WMA
Online	O	Mutual Authentication	MA

There have been modifications in the already applied protocols but some are still prone to the said attacks (Brands & Chaum, 1993; Capkun & Hubaux, 2006; Tu & Piramuthu, 2007; Nikov & Vauclair, 2008; Singelée & Preneel, 2007). That being said, some protocols lack the properties of being lightweight, computationally and processing vice controlled but secure with proper storage. We felt the need for a more secure protocol which can give security in terms of confidentiality, authentication and integrity. According to BBC News (https://www.bbc.com/news/business-49273028), in England and Wales, for the first time in 8 years, 106,000 cars were stolen in 2018. A scheme is considered safe thus, if it is resilient to:

1. Relay attack (Mafia and Terrorist Fraud)
2. Impersonation Fraud
3. Distance Fraud and Hijacking

### Contributions

The main contributions of this paper are:

• The paper provides the detailed explanation of 12 existing DB protocols. Overview of each protocol is presented.
• A new protocol is proposed, highlighting all the phases of the DB. Threat analysis and security validation is performed on the proposed scheme.
• An analysis is also presented with the help of 10 metrics to draw comprehensive comparison among existing DB protocols and our proposed scheme.
• The practical implementation of the protocol in carried out using Python.
• Areas of application and future horizons for research are discussed.

### Outline

The outline for the rest of the paper is as follows:

• It gives literature review of the many existing DB protocols as well as describes a system and threat model against many known DB protocols.
• It presents the proposed protocol in detail and explains each phase of the scheme extensively.
• It also presents a theoretical comparison of our proposed protocol against many studied DB protocols.
• It explains the security analysis and validity assumptions made for analysis of the proposed protocol.
• The performance analysis of the scheme under situation of Mafia Fraud and Replay Attack is discussed. The areas of application of the proposed scheme are presented.
• Open areas for research and future works are discussed and paper is concluded.

## Preliminaries

### Distance bounding protocols

These are cryptographic technique which allows two parties (Verifier and the Prover) to verify each other over a particular distance. The protocol has three phases namely; Initialization Phase (IP), Rapid Bit Exchange Phase (RBEP) and Authentication Phase (AP).

1. **First Step—Initialization Phase:** The protocol starts with the both the parties sending each other challenge bits (Nonces, Bit String, etc.). The next step involves both parties generating their specific bit-sequences using a function which could be either Pseudorandom Function (PRF), Hash Function, MAC Algorithm etc.
2. **Second Step—Rapid Bit Exchange Phase:** The verifier then send a bit to the prover. The prover replies with a particular bit (on the bit received from the verifier side). This is iterated “k” times (where k is pre-determined). The verifier then computes the time of the phase.
3. **Third Step—Authentication Phase:** The basic protocol in terms of authentication was studied by Bellare & Rogaway (1993); Guttman, Thayer & Zuck (2004). Nonce is generated by reader which is used to create Pseudo Random Number, using Pre—shared Pseudo Random Function and key. The whole is sent to the prover P which then verifies the string and applies the same process and sends his nonce to the reader. Both in this way authenticate each other, thus given the name “Mutual Authentication”.
  All DB protocols involving the round trip time; work on the following assumptions as presented by Desmedt, Goutier & Bengio (1987):
  • The noise delay and cryptographic operation delays should not slow down the protocol.
  • Speed of light will be used to calculate *t*_*max*_.
  • 1 bit is sent for the calculation of round-trip time.
  • During the Rapid Bit Exchange Phase, no other computation is occurring.

### Cryptographic primitives

Before digging into the protocol, there are some cryptographic functions that need to be discussed briefly.

1. **Hash functions “h”**

**Figure 1:**
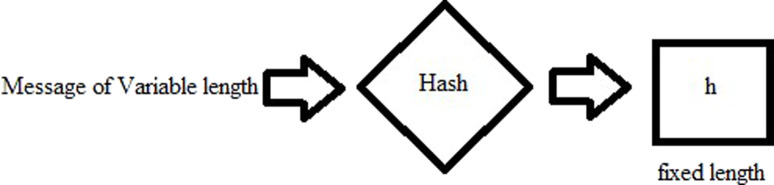
Hash function.

A variable input is given to the hash function, which converts it to a fixed length value output, which is the hash. Given that one knows the hash, it is practically impossible to obtain the input value. These makes the hashes very secure and unbreakable. More of this is given in Sobti & Geetha (2012). Figure 1 shows a simple hash function. Another property which the hash must have, is that by flipping of one bit from the input, the change in the output of the hash should be more than 50%. This is also known avalanche effect Motara & Irwin (2016).

2.**Message Authentication Code (MAC)**

**Figure 2:**
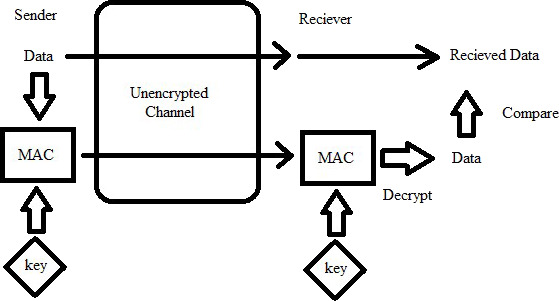
Depiction of MAC.

Message Authentication Code (MAC) Bernstein (2005) is a short piece of information, used to determine the authenticity and integrity of the message; i.e., that it came from the actual sender and that it has not been tempered with on the way. The verifier can detect changes made to the message. “Tag” is also a name given to the Message Authentication Code. Figure 2 shows MAC.

3. **Pseudo Random Function (PRF)**

Pseudo Random Function Håstad et al. (1999) as the name suggests, are functions whose all outputs are random answers (such that they are close to randomness since absolute randomness is impossible), irrespective of how the inputs are chosen. Pseudo Random Functions are not be confused with Pseudo Random Generators. The latter generates single random output for random input. The PRF generates random outputs regardless of the input given.

4. **Commitment Schemes (CS)**

This scheme allows a party to commit to a certain value, but keeps it hidden from other parties. The value can be revealed at a later stage. Once committed (as the name suggests) the party cannot change the value.

The scheme consists of two algorithms;

• Com = Commit (msg, nonce); function takes a message and a nonce as input and return a commitment.
• Verify (com, msg, nonce); function takes a commitment, message and a nonce as input and return true if values of comm matches and false otherwise.

As stated before, two properties should hold;

• Given “com”, it is computationally impossible to find the message.
• For an attacker, it is computationally impracticable to find a rogue pair (msg’, nonce’), such that; comm = comm’
  where; comm = legitimate commitment comm’ = rogue commitment

5. **Zero Knowledge Protocol (ZKP)**

It is a method in which two parties; the prover proves to the verifier, that he knows a certain value without communicating the value itself. No other information is conveyed. The challenge is for the prover to prove himself without revealing any additional information Guillou & Quisquater (1988). It is also termed as Zero Knowledge Proof.

## Distance Bounding Protocols in Literature

In DB scenario, designing an efficient scheme has always been a challenge. This is due to the fact that the proving entity in the entire model is a resource constrained device in terms of battery, storage, processing power and bandwidth. DB protocols with PKI, signatures, ECC render the scheme inefficient and impractical. We in the literature review present some of the work proposed already and their relevance to the prevailing concerns of the resource constrained part (the prover).

A modified RIFD DB protocol providing security against Mafia and terrorist fraud is evaluated by Tu & Piramuthu (2007). The RFID tag reader is vulnerable to MF or TF due to inability of reader to verify location of tag. The protocol is a concatenation of Brands & Chaum (1993), Hancke & Kuhn (2005) as well as Reid et al. (2007). Also multiple readers can decrease relay attack by triangulation.

A new RFID based protocol is introduced by Hancke & Kuhn (2005) with practical implementation and consideration of noise. The author claims that their protocol is much faster and efficient than Brands & Chaum (1993). Another novel scheme is introduced by Bussard & Bagga (2005) with implementation and security analysis. Using DB protocols to countermeasure Mafia Fraud and Observer Fraud with implementation of PKI scheme is shown in Brands & Chaum (1993).

The research Hancke & Kuhn (2005) proposed that to achieve DB resolution for RF based devices, ultra-wide band (UWB) radio is necessary. UWB devices have been used to implement DB protocol by Tippenhauer & Čapkun (2009) and Kuhn, Luecken & Tippenhauer (2010). Reid et al. (2007) proposes an alternative solution which detects the relay attack without going for the expensive UWB radio. It is the first symmetric DB protocol. The technique however is informal, and a formal definition is still an open area of research. The range of the overall system is also reduced by applying this technique which is again an open area for further study.

The concept that secret sharing scheme, based on threshold cryptography, can defeat terrorist fraud was presented by Avoine, Lauradoux & Martin (2011). Test protocol of Hancke & Kuhn (2005) was used to form two types of new protocols; Threshold DB and Thrifty Threshold DB. A protocol of the same kind is preseneted in Bussard & Bagga (2005). The same model has also been applied to *Swiss Knife* Kim et al. (2008). A new protocol with heightened security and lightweight nature was introduced by Guttman, Thayer & Zuck (2004). It showed how both the protocols *Swiss Knife* Kim et al. (2008) and Avoine, Lauradoux & Martin (2011), are resistant to DF and MF; but susceptible to the new Hancke—TF attack Hancke (2012).

Different attacks on Kim & Avoine (2011), Kim et al. (2008); showing attacks such as relay attack, terrorist fraud, mafia fraud as well as dictionary attack in ideal and real life communication channel is presented by Peris-Lopez et al. (2009) General design guidelines are given for designing a secure and effective DB protocol.

For the first time, integrity and privacy was introduced by Waters & Felten (2003) where latency of the round trip was measured. They introduced the concept of location manager; authenticated with Public Key Infrastructure (PKI). They approved exact location of device even when it is held by adversary. In practical application, deployment of the protocol faced issued due to physical factors and ownership drawbacks. The protocol actually traded off security for location proving. Another innovative protocol was presented by Nikov & Vauclair (2008) where pre-processing is non iterative and lighter. Symmetric techniques were used, with the use of authenticated nonce which increased the overall efficiency and relaxed the resource requirement for the prover.

The introduction of void challenges is introduced in Munilla & Peinado (2008). It is a challenge where the reader leaves deliberately to check whether an adversary is trying to get the response from the card in advance. The protocol of Hancke & Kuhn (2005) was used as basis and then the protocol was modified using the void challenge technique. This decreases the adversary’s probability to access the system. Analysis in noisy case, Bit Error Rate (BER) and false alarms is also conducted. The author state that the proposed protocol works better than the original. Singelée & Preneel (2007) presented a low cost DB protocol for noisy environments, which uses binary codes in the rapid exchange phase, to correct bit errors.

Capkun & Hubaux (2006) dealt with the problem of positioning is wireless systems. Position and Distance Spoofing attacks were conducted on positioning techniques to check their resistance. They proposed a mechanism for securing position in wireless devices and sensor networks and verified the same with simulations. Avoine & Tchamkerten (2009) proposed a low complexity protocol without compromising the performance of the scheme. In their protocol, the verifier has the choice to accept or reject the message of identity even if the protocol in halted in between an ongoing session. A survey highlighting the security of the DB protocols has been carried out by Avoine et al. (2018) and Brelurut, Gerault & Lafourcade (2015), which tells about the different attacks on 23 different DB protocols, countermeasures, and methods of analysis pointing out that cluster based comparison can be modified for better practicality.

A set of mechanisms for multi node wireless networks that give secure verification of time encounter is introduced by Čapkun, Buttyán & Hubaux (2003). It was based on Merkle Hash Tree and one-way hash. They problem of securing topology and tracking was addressed by them for the first time. They introduced mitigation to wormhole attack, securing the routing protocols and cheating detection by topology tracking. Incorporation of challenges for mutual authentication were also proposed. However, their scheme lacked any verification by software simulations and thus, is still an open area for future studies.

Kardaş et al. (2012) introduced Physically Unclonable Function (PUF) which is a digital fingerprint. This protocol introduced a very strong adversary having the power to access the tags’ volatile memory. It proved that Kardaş et al. (2011) is not safe according to this model. The use of PUF enhances the security and privacy of the protocol, making it cost effective. The use of signature provides ideal security against TF. Tuyls & Batina (2006) presented PUFs to store key and for this, public-key cryptosystems were used. As all the keys are fabricated at different interval, therefore whole secret key cannot be extracted from the tag. The protocol provides security against TF, MF and DF, which can be further increased by addition of signature in the last stage of the protocol. It is the first research with (1/2)n security against all frauds.

The process to avoid relay attacks during authentication was given by Avoine, Floerkemeier & Martin (2009). The RFID not only reduces the success probability of the adversary, but also decreases the rounds executed within the protocol. A unified framework for RFID DB protocol is presented by Avoine et al. (2011). White and Black Box models are presented. Test procedure of Munilla, Ortiz & Peinado (2006) is used. The framework can be altered to analyze or design DB protocols.

SKI was introduced, which is first family of lightweight and provably secure DBP, by Boureanu, Mitrokotsa & Vaudenay (2015). These are secure even under real time scenarios. Countermeasure against TF and MF are secret sharing with leakage scheme and circular keying with Pseudo Random Functions (PRF). PRF is also used in reuse of keys and to fix common security claims. Further improvements can also be made to enhance the design and to assure resistance to TF in occurrence of noise. The study that SKI can resist all frauds was given by Boureanu, Mitrokotsa & Vaudenay (2013b). It claimed that SKI is the first scheme with all accompanying security pledges.

Distance Hijacking attack on 19 protocols was studied by Cremers et al. (2012), with countermeasures, modelling and formal analysis. It also paves way for future research including addition of privacy preservation and protecting location privacy. A new type of relay attack was proposed by Wei, Zhang & Wang (2016), which launched the spoofing attack within an effective distance range. The problem was rectified by using time stamping verification; which verifies the efficiency and corrects certain flaws in the protocol. A state of the art distance bound model with three parties (the third being the hardware) has been proposed by Kılınç & Vaudenay (2018). The model is called Secure Hardware Model (SHM), in which the prover has the hardware but cannot access it fully. A new protocol is given in sync with the proposed hardware model.

Assuming that the information established from prover can be replayed to launch a terrorist fraud was proposed by Avoine et al. (2017). Basic construction for provably secure distance bounding protocols was presented with symmetric key, public key anonymous protocol. Bussard (2004) proposed anonymity of the prover by the help of a dedicated scheme, which is an extension of group signatures. Proof of knowledge scheme was applied with cryptographic and distance measuring techniques. A framework for establishing trust based on history was implemented. In other works, Privacy and information leakage was studied by Rasmussen & Čapkun (2008). The concept of three verifiers was introduced by Capkun & Hubaux (2005), Shmatikov & Wang (2007), Singelee & Preneel (2005). Collision attacks were studied by Chandran et al. (2014), Chiang, Haas & Hu (2009).

The study of distance bounding was studied in RFIDs Drimer & Murdoch (2007) and sensor networks by Meadows, Syverson & Chang (2006), Capkun & Hubaux (2005). Electronic equipment was used to execute distance bounding protocols Rasmussen & Capkun (2010). A new DB protocol was proposed by Sastry, Shankar & Wagner (2003). The protocol is based on Ultrasound and wireless radio communication, and can only be used to verify the position of the nodes. Mitigation of wormhole attack was proposed by a new mechanism “Packet Leashes” by Hu, Perrig & Johnson (2003). A mechanism for securing against spoofing attack has been proposed by Kuhn (2004). The reliance on long term shared secret is exempted. Another scheme has been proposed by Meadows et al. (2007) which uses only a single round in rapid bit exchange phase.

Avoine et al. (2021) proposed DB protocols as the main countermeasure against relay attacks. Relaying mechanism, threat models and some pivotal challenges in Distance Bounding domain are discussed in detail. Abidin (2020) proposed use of Qubits to detect relay attacks against RFID systems. All DB protocols; according to them; are based on traditional crypto-graphical techniques. They found a weakness on the protocol of Jannati & Ardeshir-Larijani (2016). In this loophole, attacker with Quantum Memory can easily mount a relay attack. A new countermeasure is proposed and then compared with the original.

Cryptographic Protocol Shapes Analyzer (CPSA) is used to classify DB protocols by Rowe, Guttman & Ramsdell (2020), taking into account the physical distance. Assumptions are made and a comparison is made on the relative strength of various DB protocols. A new symbolic model is proposed by Debant, Delaune & Wiedling (2020) in the domain of NFC. The concept of automated verification is given by the use of ProVerif tool.

## System and Threat Model

**Figure 3:**
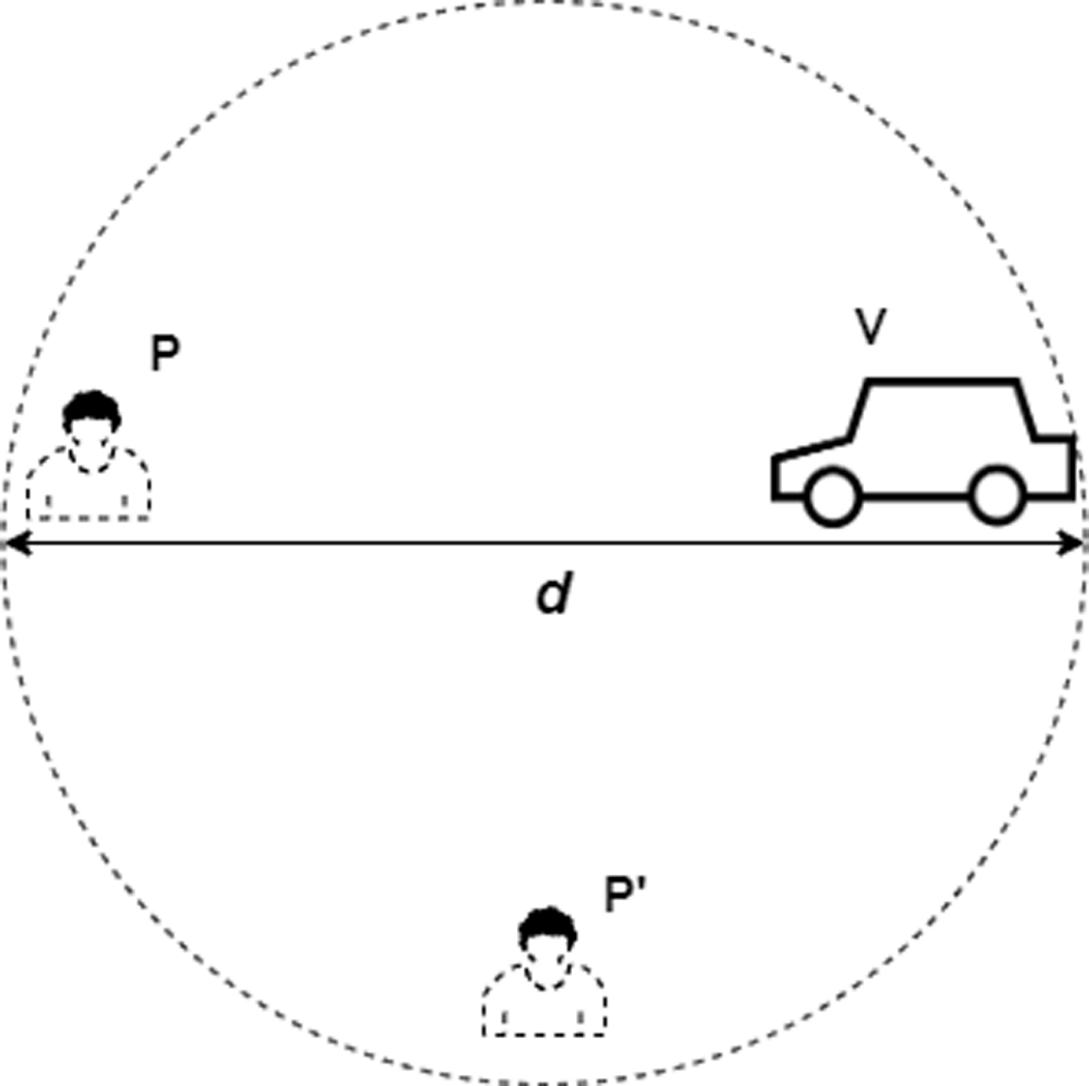
System model.

The attacks on DB protocols are namely Relay Attack, Mafia Attack, Terrorist Fraud, Impersonation Fraud, Distance Fraud, Distance Hijacking, Man in the Middle Attack, Replay / Playback Attack, Node Capture Attack and De-Synchronization Attack, etc. and are discussed in Brands & Chaum (1993), Avoine et al. (2011), Boureanu, Mitrokotsa & Vaudenay (2013b), Peris-Lopez et al. (2009), Boureanu, Mitrokotsa & Vaudenay (2013a), Avoine, Lauradoux & Martin (2011), Bussard & Bagga (2005), Kim et al. (2008), Kim & Avoine (2011), Reid et al. (2007). The system model consists of 2 entities like Prover P and Verifier V, where a malicious entity P’ is acting as man in the middle to launch any of the above mentioned attacks and gets itself authenticated within a specific range *d*, as shown in Fig. 3. The details of above mentioned attacks is given as follows:

1. ** Mafia Fraud (MF):**

Given in Desmedt, Goutier & Bengio (1987), Desmedt (1988), an adversary comes in between prover and verifier. Efforts are undertaken so that verifier accepts adversary as the prover, taking advantage of the actual prover’s position.

2. **Impersonation Fraud (IF):**

An opponent tries to masquerade as the legitimate prover, and tries to get access from the verifier.

3. **Man in the Middle Attack (MIM):** It is generalized mafia and impersonation fraud Boureanu, Mitrokotsa & Vaudenay (2013b). The goal of the attacker is to make the verifier accept the false prover with the key *“x”*. (Key *x* is known to the attacker). Man in the middle attack (MIM) is initiated by a malicious rival between real reader R and tag T. False reader R’ interacts with real tag T, and vice versa. The honest reader R to think it is communicating with the actual tag T while in real, it is connected with rogue tag T’. However, since a tag cannot be impersonated, this type of attack is only possible when the tag is cooperating with the adversary.

4. **Terrorist Fraud (TF):**

It is an extension of Mafia Fraud Attack. With the help of an adversary, the malicious prover gains access via the verifier, but the adversary alone cannot get the access. The Tag T is not legitimate and uses a rogue tag T’ to convince reader R of its location. The attack becomes possible when the tag reveals its secret key to the adversary.

The way to prevent this attack is such that the Rapid Bit Exchange Phase is amalgamated by means of cryptographic primitives and schemes. The protocol cannot be split into two discrete segments by the rival. This can be accomplished by use of confidential hardware and / or use of well secured private (or symmetric) key during RBEP.

5. ** Distance Fraud (DF):**

An illegitimate prover; at a certain distance; tries to get access form the verifier.

6. **Distance Hijacking (DH):**

A malicious prover, at a distance, tries to take advantage of the legitimate provers to get access from the verifier.

7. **Node Capture Attack (NCA):**

Legitimate nodes are physically captured by attacker to extract vital information from them. The attacker can then make his own node clone or use that information as per his will. More examples can be found in Lin & Guowei Wu (2013), Tague & Poovendran (2008), Strasser, Danev & Čapkun (2010)

8. **Mutual Authentication (MA):**

Both reader and tag get the conviction that they are communicating with the claimed legitimate entity. (Reader in case of Tag; Tag in case of Reader).

9. **Relay Attack:**

Given in Silberschneider, Korak & Hutter (2013), the attacker only relays messages between two parties. The attacker may or may not read or influence messages. The mafia and terrorist fraud are types of relay attacks.

10. **Replay Attack / Playback Attack (PA):**

It is also called playback attack. It can be easily described as an inferior version of Man in the Middle Attack (MIM). The attacker re-transmits legitimate data as per his own choice.

11. **De-synchronization Attack (DSA):**

Lo & Yeh (2010) attack on the RFID system in which the shared key of verifier and prover does not match. This happens because of an attacker jamming the communication.

**Figure 4:**
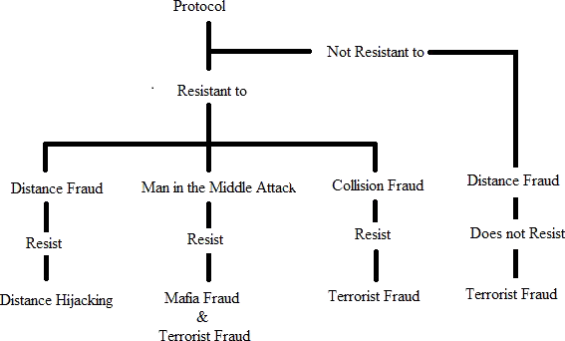
Brelurut theorem.

Brelurut, Gerault & Lafourcade (2015) presented the following theorems which are also presented in Fig. 4.

• DF − > DH: A protocol resilient to DF is unaffected by DH.
• MIM − > MF and IF: A protocol resilient to MIM attack is resilient to MF and IF.
• CF − > TF: A protocol resistant to CF is also resistant to TF.
• DF − > TF: A protocol non- resilient to DF is non-resistant to TF, with a better success probability.

## Proposed Protocol

The basis of our protocol is formed on the ideas taken from various renowned protocols; MAP1 (Bellare & Rogaway, 1993), MAP1.1 (Guttman, Thayer & Zuck, 2004), protocols of Kim et al. (2008), Hancke & Kuhn (2005) and Munilla & Peinado (2008). The introduction of symmetric schemes, pre—shared main and transient keys; as well as a bit string are major amendments. The goal of our proposed protocol is mutual authentication (authentication of both the verifier by the prover and vice versa) achieved while running on low computational cost. The DB Protocol is unlike other authentication protocol due to its need for low processing and constrained resources. The protocol as described before can be divided into three distinct steps. The components for better efficiency and strength will be discussed for each step.

The first step namely the Initialization Phase takes a bit string, applies a certain cryptographic function on it and passes it onto the second entity where again, the same cryptographic function in applied on it. The strength of this phase depends upon the strength of the cryptographic function used which is a trade-off between hash, MAC, PRF etc. For simultaneously addressing the need of security and performance, we have opted not to use PKI or Signatures in the first phase, rather we have introduced two separate pre-shared secrets. In this way, even if one key is compromised, the entire protocol is still safe. The intermingling of the bit string is carried out by the use of Pseudo Random Function (Hash or MAC). The bit string size is also taken as 512 bits as to add complexity to the protocol.

The second step namely the Rapid Bit Exchange Phase is the communication of the result of the cryptographic function (of the first step) between the two parties. This step is more of a challenge –response phase where a verifier sends a bit and gets a response based on the bit sent. The responses are saved for the third and the last phase. The time of this phase is also recorded. In contrast to existing DB protocols, it ensures added security such that even if the bits sent and received are sniffed and relayed by any adversary, the time factor and the pre-shared sequences at both ends limit the probability of many attacks to get successful. We will prove all claims about enhanced security of our protocol in the Security Validation Section.

The third and last phase namely the Authentication Phase is the phase in which the verifier checks the responses sent by the prover. Error is checked on the received corresponding bits and based on a threshold, access is either granted or denied. The time of the second phase recorded is also checked with previous value. The reason for checking the time is that the prover needs to be close to the verifier i.e., within a specific range (usually few meters) at the time of seeking authentication. This becomes an indirect form of authentication and also caters for Relay and Replay attacks on its own. The strength of this phase thus depends upon the error checking protocol and the time checking algorithm. Some protocols (already studied in the literature) have proposed Error Correction Techniques, which in our opinion cause overhead on the processing and computational power, and cause certain delay in time as well.

Our proposed protocol uses minimal computational power and resources, excluding the need to use Public Key Infrastructure (PKI) and various other heavy encryption standards. The protocol uses two pre-shared secret keys *“x”* and *“m”*; uses Pseudo Random Function (PRF) in the Pre-Computational and Initialization Phase. A security parameter or bit size of 512 is taken in the protocol. The step wise protocol is discussed in detail below.

### Pre-computational phase

**Figure 5:**
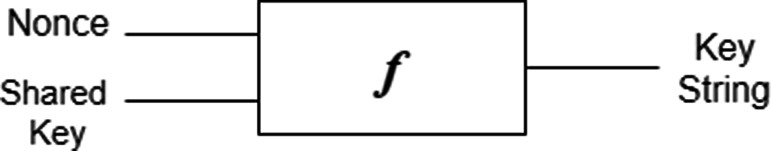
Derivation of bit string.

Both Verifier and Prover generate random nonces “*N*_*v*_” and “*N*_*p*_” respectively. These nonces are used to generate k-bit strings *“a”* and *“b”* on the prover and verifier side respectively. The prover uses the shared secret *x* while the verifier uses shared secret *“m”*. The cryptographic function used for derivation at both ends is shown in Fig. 5. This function can be a trade-off between hash, MAC, PRF etc. based on available resources.

### Initialization phase

Both parties send their nonces to each other. They use the nonces of each other to generate *“b”* and *“a”* at the prover and verifier side respectively. the same cryptographic funtion is used as shown in Fig. 5. Only this time the prover uses the shared secret *“m”* while the verifier uses shared secret *“x”*. The verifier chooses a k-bit random sequence *α*_*i*_.

### Rapid bit exchange phase

The verifier starts the timer and sends first bit of the *α*_*i*_ to the prover. The prover replies with his response *β*_*i*_ in the following manner:

If *α*_*i*_ = 0 ; *β*_*i*_ = *a*_*i*_ and; if *α*_*i*_ = 1; *β*_*i*_ = *b*_*i*_

Although the value of *α*_*i*_ is random, the prover will be on the lookout for a specific sequence of bits, e.g.; 1011. After completion of this sequences 1011, the immediate next bit of *α*_*i*_ will determine the response from the prover. The replies will be reversed.

If *α*_*i*_ = 0; *β*_*i*_ = *b*_*i*_ and; if *α*_*i*_ = 1; *β*_*i*_ = *a*_*i*_

The rest of the protocol will follow as usual. The significance of this sequence is that it allows the prover to verify the verifier without putting computational load on the protocol. In case, where mutual authentication is not required this part can be replaced with the simple relaying of a challenge bit *α*_*i*_ from the verifier, resulting in a response bit *β*_*i*_ from the prover’s side.

### Authentication phase

The verifier will verify the responses *β*_*i*_. He will compute the error in the transmission, which includes checking the received value of *β*_*i*_ with the pre-computed one. For instance, let *β* be the bit received after the RBEP and *β*′ be the original (intended) values (pre-computed on the verifier side). Error will be calculated as:

err *β* = count *β*_*i*_! = ${\beta }_{i}^{{^{\prime}}}$ and Δ *t*_*i*_ > *t*_*max*_

where; *β*_*i*_ = response bit ; ${\beta }_{i}^{{^{\prime}}}$ = pre-computed bit If the value error is greater than a pre–computed threshold “T”, then the process will be terminated. For argument’s sake, if at the end of the protocol, the verifier has all the values of *β*_*i*_, transmitted by the prover in RBEP. He computes error of each individual bit, by comparing each transmitted *β*_*i*_ with his own computed values of *β*_*i*_. Error percentage is computed. If the error exceeds a pre-defined threshold, the protocol is stopped. The mathematical representation of the protocol is given in Table 2.

**Table 2:** Proposed Protocol.

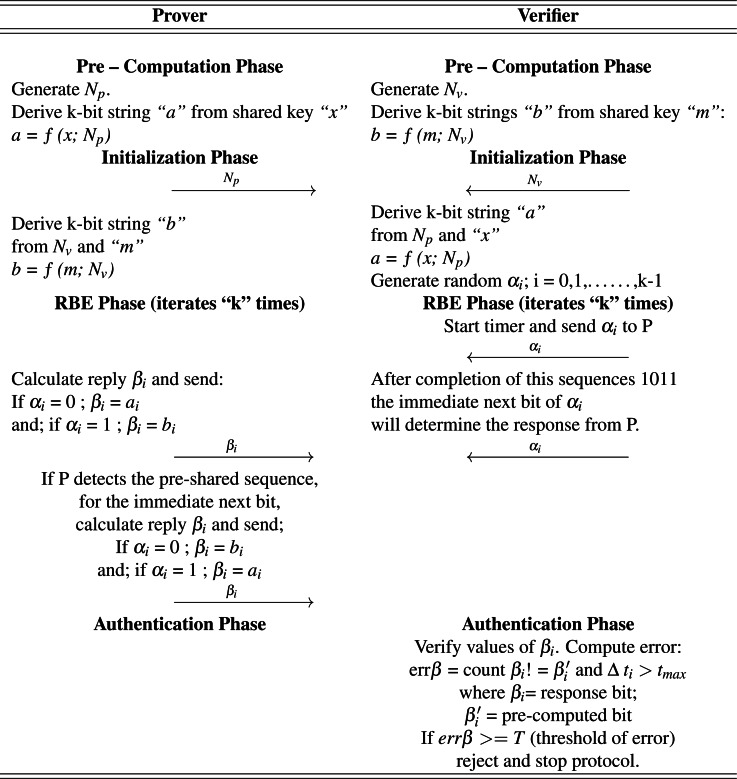

### Correctness of proposed protocol

Our proposed protocol consists of two entities i.e., Prover P and Verifier V having their own secret keys *“x”* and *“m”* respectively. Before the formal start of protocol, the nonces are generated and fed into a cryptographic function along with secret keys as another input to derive 2 different 512-bit length strings, one on each side. After the protocol initiates, nonces are shared between both parties and the opposite bit strings are derived using the same function but with the opposite entity’s secret key as the second output.

Since the cryptographic function is already known and nonces are shared over wireless media, an adversary can sniff / or capture these nonces and use them to derive bit strings used during RBEP if and only if he has both the secret keys. Keys are pre-shared and are never shared publicly. Since both entities know both the bit strings, it’s very easy for verifier to generate a random bit sequence and send it as challenge to prover and for prover to respond with the correct bit. Verifier checks for the authenticity of the prover’s response by verifying values with his own and computes error. In this scenario, since both entities have the same sequences at their end i.e., all the values of *a*, *b*, *α*_*i*_ and its corresponding *β*_*i*_, it’s very easy for the protocol to run its course smoothly and prover gets authenticated and is granted access.

### Comparison with existing DB protocols

The protocol was analysed for attacks and error resistance. Any errors faced during the RPEP are detected. The last entry in Table 3 compares our proposed scheme with the rest of the literature.

**Table 3:** Comparison of Existing DB Protcols.

S. No.	Protocol	Security	Pre-processing capability	No. of phases	Cryptographic primitives	Defence against	Vulnerable to	Resistance to channel errors	Privacy against attacks	MA	Total computation (both sides)
1.	Capkun & Hubaux (2006)	b	h	3	No	MF	TF & NCA	Yes	N/A	Yes	2(Commit)+2(MAC)
2.	Waters & Felten (2003)	N & X.509	No	3	Yes “S”	N/A	DOC & NCA	N/A	Yes	Yes “IDs”	4(PKI)
3.	Brands & Chaum (1993)	n	No	3	No	MF & TF	TF & NCA	No	N/A	No	2(BPKI)
4.	Hancke & Kuhn (2005)	s	h	3	No	MF	TF & NCA	Yes	N/A	No	2(OWCRHF)
5.	Bussard & Bagga (2005)	k	No	3	Yes	MF, TF & DF	N/A	Yes	Yes “S”	Yes	1(PKI)+1(ZKP)
6.	Reid et al. (2007)	x	PRF	3	No	MF & TF	NCA	Yes	No	No	2(PRF)+(SE)
7.	Nikov & Vauclair (2008)	R	Yes	3	No	MF	TF & NCA	No	N/A	No	4(PRF)+2(HMAC)
8.	Munilla & Peinado (2008)	x	PRF	3	Yes “S”	RA	NCA	Yes	N/A	Yes	n(h)
9.	Singelée & Preneel (2007)	(n,k)	No HD	3	No “ECC”	MF	TF & NCA	Yes	N/A	Yes	4(ECC)+2(MAC)
10.	Tu & Piramuthu (2007)	L	h GSK	3	No	MF & TF	TF (SK) & NCA	No	N/A	Yes K(Temp)	4(h)
11.	Kim et al. (2008)**MA**	s	PRF	4 MAP	No	MF, TF & RA	NCA	Yes	Yes	Yes	3(PRF) (1 PC)+(2 O)
12.	Kim et al. (2008)**WMA**	s	PRF	3	No	MF, TF & RA	NCA	Yes	Yes	No	2(PRF) (1 PC)+(1 O)
13.	Avoine & Tchamkerten (2009)	s	PRF	3	No	MF & TF	NCA	Yes “DT”	N/A	No	2(PRF)
14.	**Proposed scheme**	k	PRF	3	PRF & h	MF, TF, DH & DF	NCA	Yes	Yes	Optional	2(PRF)

Our proposed protocol has privacy preservation from outsiders and does not reveal secret keys to the attacker. The possibility of a Man in Middle Attack exists where the attacker can only sniff the traffic, but cannot relay it over a long distance because that would cause delay in propagation time. The total computation involves the use of two PRFs, one on each side in the Initialization Phase and Error Check towards the verifier side in the Authentication Phase. The comparative performance and security analysis of the twelve well known protocols were carried out for a better understanding and working of the overall DB protocol. The conclusion drawn were used to strengthen the protocol that we designed in this study.

The proposed protocol as evident from Table 3, has numerous benefits over other DB protocols. Our proposed solution has capability of pre-processing, which means that the initial generation of the nonces and the encrypted numbers “*a*” and “*b*” can be carried out beforehand, which reduces the resources for on chip processing. It uses hashes and PRF which are secure for less complex key phrases for a longer time without compromising the resource need. The protocol is resistant to most forms of attacks and channel errors. The total computation is of the scheme is 2(PRF) + (EC). The notion of Mutual Authentication is optional as we feel that this will burden the protocol and make it impractical. However, this may pave way for further research and future studies.

An theoretical analysis given in Table 3 indicates the numerous functions used in the phases. It also presents the computational time taken by the protocol, the use of PKI, attack possibility and resistance, reason for possibility and / or success of the attack. The comparison is based on several parameters; security dependence, pre-processing capability, cryptographic primitives used, defence and vulnerability to known attacks, error resistance to channel error, privacy preservation to outsiders and total computational cost of the entire protocol (based on the constrained functions used) of each scheme. These all together make up a total of 10 common metrics, which are carefully chosen after the study of various DB protocol.

## Security Validation

This section presents the security validation of the DB Protocols and explains how our proposed protocol is secure against the possible threats. The scheme is checked against all attacks discussed before in System and Threat Model Section.

### Assumptions

Before the analysis of the protocol, the following assumptions are made:

• The legitimate Prover and Verifier are denoted by P and V, while the rogue Prover and Verifier are denoted by P’ and V’.
• Statistical Attacks like brute force attacks are possible even on the most secure encryption standards like DES and AES. We will not explain them in much detail as it falls out of the scope of this research. Also, for *k* = 512, there exists 2^512^ combinations for *α*_*i*_. Brute forcing the RBEP with each combination is impractical and useless for the assailant.
• The pre-shared secret is only present with the verifier and the prover and there is no way for an attacker to extract them other than by means of a Node Capture Attack (NCA).

###  Security against threat model

1. **Mafia Fraud (MF)**
  Since it has been established before that the only way to achieve full protection against mafia fraud attack is to either use PKI or Zero Knowledge Protocol. Both of them are computationally heavy and therefore cannot be applied in this domain. Thus, a clever approach is needed.
  In a scenario where Actual Prover P and Verifier V are not close to each other, it is impossible for P’ and V’, to relay messages between them without considerate delay (which would cease the protocol). The use of random *α*_*i*_ also prevents replay attack.
  ***Pre computation and initialization phase***
  As the function used in Pre Computation and Initialization Phase is pseudo-random, therefore guessing of any bit of *“a”* and *“b”* by the attacker is negligible. The probability is further minimized by the use of different keys for *“a”* and *“b”*. This further achieves randomness.
  ***Rapid bit exchange phase***
  For the Rapid Bit Exchange Phase, the Actual Prover “P” looks for a specific sequence (for example: 1011). When this sequence is completed, in the next consecutive bit only, the reply from the Actual Prover P is reversed (as seen in the protocol).
  ***Authentication phase***
  If in any case if the attacker is using his own pair of *a’* and *b’*; then the probability that he will send the specific sequence of *α*_*i*_ in RBEP is very low i.e., lower than (1/2) n as depicted by Hancke & Kuhn (2005)), and becomes even lower due to use of random *α*_*i*_ in each RBEP. The probability becomes even lower than he can respond correctly i.e., send correct *β*_*i*_ with the order reversed. The checking of corresponding bits by the verifier V and calculating error further reduces the attackers’ chances. The same can be applied to **Impersonation Fraud (IF)** and **Man in the Middle Attack (MIM)**.
2. **Terrorist Fraud (TF)**
  To prevent this attack, the Rapid Bit Exchange Phase (RBEP) are mingled by means of cryptography. The protocol cannot be split into two discrete segments by the rival. This can be accomplished in two ways i.e., to use confidential hardware and / or to use well secured private (or symmetric) key during RBEP.
  Both of these steps are computationally constrained and slows down the protocol. In simple words, the attacker should not be able to achieve the information that the Actual Verifier V holds, which is the shared key “*x*” and transient key “*m*”.
  In case of our protocol, let actual Prover P be far from the actual Verifier V and close to the rogue Prover P’. He relays the pair *a*_*i*_ and *b*_*i*_ as per the protocol to the Rogue Prover P’, independent of the value of *α*_*i*_ (as the actual Verifier V isn’t close by). If the verifier is close by, then the attack becomes replay attack. Even if P’ possess all the values of *a*_*i*_ and *b*_*i*_, and relays it to the actual Verifier V, even then guessing the shared secret “*x*” and “*m*” is impossible because:
  • The probability that the pair *a*_*i*_ and *bi* are in sync with *α*_*i*_ while communicating with V is very low (very less than (1/2) n). Also, for 512 bits, the combination of *α*_*i*_ becomes 2^512^. This means that guessing the sequences is near to impossible.
  • Even if some of the values do go in sync with the challenges, they will not be able to sustain the error threshold, and will be filtered. This will also only give very less and ineffective information regarding the shared secrets.
  • Both functions encrypting the nonces use a separate key *“x”* and *“m”*.
  • Independently *“a”* and *“b”* cannot be used to obtain the information that the attacker seeks.
  • As the Verifier V does not reject the value of *β*_*i*_, therefore the *i*th position remains secret to the attacker.
  • The prover will be on the lookout for the specific sequence which will be used by the entity as a way of authenticating the verifier. Given that this sequence is not received in the entire Rapid Bit Exchange Phase, it will raise suspicions to the prover. If the prover’s reply is not reversed, this will alert the verifier.
3. **Distance Fraud (DF)**
  For the attacker to execute a distance fraud attack, the value of *β*_*i*_ should be responded in advance by the rogue Prover P’, for which he needs to choose the response at random and send it to the Actual Verifier V.
  The probability that the “*β*_*i*_” chosen by the P’ is matching the actual *β*_*i*_ is very low; keeping in mind that not only are these values pseudo-random, but also use different keys for randomness. The reversing of *β*_*i*_ in the Rapid Bit Exchange Phase further minimizes the chances of a match. If the value of *α*_*i*_ bits for the RBEP is 512, then the combinations possibly become 2^512^. Guessing this many combinations is impossible for the rogue Prover P’ in the given time without much delay. The error checking in the Authentication Phase will render the attack useless. Also, if it is resistant to DF; therefore according to Brelurut, Gerault & Lafourcade (2015), it is secure against **Distance Hijacking (DH)** as well.
4. **Node Capture Attack (NCA)**
  The threat of theft is possible in all devices. The tag or reader; if stolen can be read by a chip / RFID reader and necessary information can be extracted. Therefore, the following measures should be taken:
  • Keep the verifier (reader in case of an RFID) in a secure place. For example, in a key less entry system used in vehicles, the reader (ECU) is inside the vehicle in the dashboard and is well protected by lock and key. If a malicious entity gains entry into the vehicle, even then accessing reader (ECU) is a difficult task, usually involving the breakage of the dashboard panel at the back of the steering wheel.
  • Keep the prover (tag in case of RFID or key fob in case of keyless entry system) on your person while in the vicinity of the verifier (reader). The tag should be kept secure even when not in use.
5. **Mutual Authentication**
  The verifier authenticates the prover in the last phase (Authentication Phase) where the error is computed, time of the protocol is checked and access is either granted or denied.
  Older schemes either lacked mutual authentication; or the ones that did not, involved the use of signatures which made the protocol computationally heavy or impractical.
  In our employed scheme, the prover is on the lookout for a specific pre-shared sequence that actually authenticates the verifier. This provides only Pseudo –Authentication as the sequence can come in any of the combinations, but even then, it is better than having no authentication at all. This provides double verification of the prover as well as the response of these sequences in the later phase authenticates the prover.
6. **Relay Attack**
  It is impossible for P’ and V’ to relay messages between themselves and towards the legitimate parties without considerate delay where any delay above a certain time limit will cause the protocol to terminate. There is also a chance of error during transmission. The time of the RBEP Δ*t*_*i*_ is checked in the Authentication Phase i.e., Δ*t*_*i*_ = < *t*_*max*_ (standard time for protocol run).
7. **Replay Attack**
  Take an example where Actual Prover P and Actual Verifier V are close to each other, and an attacker posing as MIM. He can capture the values of *β*_*i*_ and then replay them at a later time. This issue is resolved in the RBEP. The value of *α*_*i*_ is random and therefore the response which the verifier wants is also random (response *β*_*i*_ depends upon the value *α*_*i*_). There is a probability of 0.5 that the first value of *α*_*i*_ may match (either 0 or 1). But this probability becomes unimportant with the bit size of 512, the combinations of *α*_*i*_ being 2^512^, and as we move from the second bit to the last (512th bit).
8. **De-synchronization Attack**
  The chances of De-synchronization Attack are very less because:
  • The keys are pre-shared, not updated and remain the same.
  • The distance involved is very less (few meters).

## Performance Analysis

The simulation of the protocol was executed on Intel (R) Core i5-3230M CPU @ 2.60 GHz having 10GB RAM and running Windows 10 on a 240GB SSD using Python language on Python 3.7.5 version. The protocol uses libraries of hmac and hashlib. Keyed hash message authentication code (HMAC) is used to encrypt the nonces with the respective secret key, with a SHA-512 Hash Function. The protocol takes a total run time of around 20.8 µs. Table 4 shows the run time taken in microseconds by every stage of our proposed protocol.

**Table 4:** Run time of proposed protocol.

Stage	Time (µs)
Initialization Phase	0.09
RBE Phase	20.1
Authentication Phase	0.075
Total Time	20.84

Table 5 shows the comparison of the protocol in 2 different attack scenarios. Replay attack is initiated on the protocol where bits transmitted are stored and replayed in the next run of the protocol. The received value of *β*_*i*_ as stated before, is self-given to launch the attack.

**Figure 6:**
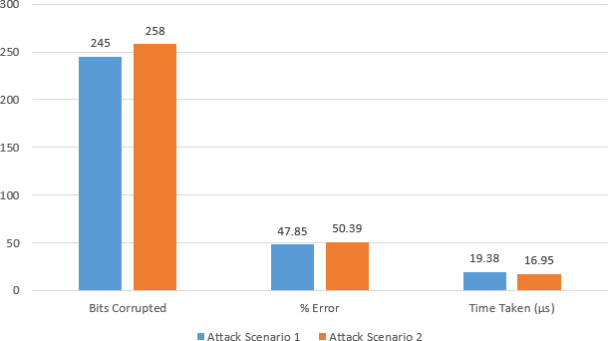
Bar graph of comparison between attack scenarios. .

In case of attack scenario 1, we launched Replay Attack and captured the entire value of *β*_*i*_ from the last run of the protocol’s code which is used as received *β*_*i*_ in the next run. The percentage error as shown comes out to be 47.85% with error count at 245 bits out of 512. This means that nearly half of the bits are incorrect. In case of attack scenario 2, Mafia Attack was launched on our protocol and the error percentage rose to 50.39% with error count at 258 bits out of 512. The run time of both these scenarios is less than the total run time of our proposed protocol as under attack, the protocol ceases to run and is terminated as soon as error threshold is crossed. This shows that our proposed scheme is resilient to any change in the bits and is able to detect the error with greater efficiency. The bar graph of comparison is presented in Fig. 6.

**Table 5:** Comparison of protocol in attack scenarios.

Parameters	Attack scenario 1	Attack scenario 2
Total bits	512	512
Bits Corrupted	245	258
% Error	47.85	50.39
Time Taken (µs)	19.38	16.95

Our proposed protocol has the capability of pre-processing which means that the initial generation of the nonce and the encrypted numbers “*a*” and “*b*” can be carried out beforehand. This saves run time as well as resources. The protocol offers defence against most of the attacks and is resistant to channel errors. It also preserves the privacy of the protocol. The total computation of the scheme is 2(PRF) + (EC). On the other hand, the shortcomings of our proposed protocol include its vulnerability to noise errors, node capture and de –synchronization attack although the chances of latter are very less. Error correction code is not applied due to computational and cost overhead. The protocol can only detect the error. It lacks the capability of correcting them.

To cater for noise errors, one of the possible solutions is to increase the number of rounds in the Rapid Bit Exchange Phase, keeping the time factor below the required delay threshold T. Another solution is to divide the number of bits of *α*_*i*_ into smaller chunks. If the bit size of the *α*_*i*_ is 512 bits then we can make 8 chunks of 64 bit each. The value of error (err *γ*) for each individual chunk will be checked and then percentage correctness can be calculated. Acceptable error should be no more than 38%.

### Areas of application

Distance Bounding Protocols find their applications over a lot of technologies. The practicality of these application has spread after the rise of newer techniques like NFC, Contact-less payment, Key less Entry Systems, Ticketing, RFIDs, entry and exit at a specific point, attendance of employees via tags, access to systems (like computer) via tags, etc. The list is by no means exhaustive and can be further explained.

For our study, we restrict ourselves to modern key less entry systems used in vehicles specially cars nowadays, where the distances taken into account are not that large. That being said, the danger of malicious entry is still there and becomes somewhat of paramount importance given the fact that the entry system in the vehicle authenticates the credentials of the person; but not the person himself.

This becomes a bigger issue in advanced systems when the need to place hand on the sensor for authentication is removed. The car authenticates the user / driver (prover) as soon as he is in the vicinity of the ECU (which in this case, is the verifier). Thus, the need for a secure system becomes perilous in these situations.

## Conclusion and Future Areas of Research

### Conclusion

DB protocol enables validation of two entity validation over a distance. While this offers ease and technological superiority, it also raises security concerns with it. This paper has listed some of the security requirements for an efficient and attack resilient protocol.

The literature review of several protocols in practice has been carried out, highlighting the bit exchanges and attacks possible on the DB scheme. Furthermore, a novel protocol is proposed which offers low computational and is resistant to most attacks. The claim is validated informally; by threat modelling and formally; by software analysis. Results for protocol run is different time scenarios are also presented.

### Future areas of research

For the future, we would like to do the analysis of the protocols in literature, using a standard or a software realization (for this purpose mathematical modelling can be applied or a NIST standard for stream ciphers can be used). We would also like to verify the proposed protocol on a software tool other than used here.

Error Correction Code and hardware implementation of the protocol are also open areas of research. The best possible features can be extracted to make an even more vigorous, unique protocol. Our proposed protocol is still prone to Node Capture and de-synchronization attacks although the chance of occurrence of the latter is much less; still, it is an open ground for future studies and research. With that being said, various privacy and efficiency requirements are being studied in other research ventures, which can be incorporated in our work as well.

##  Supplemental Information

10.7717/peerj-cs.517/supp-1 Supplemental Information 1 Code in python language.

10.7717/peerj-cs.517/supp-2 Supplemental Information 2 Explanation of Python code of Proposed Protocol.

## Additional Information and Declarations

### Data Availability

The following information was supplied regarding data availability: The python code of the proposed protocol is available as a Supplementary File.

## References

[ref-1] Abidin A (2020). On detecting relay attacks on RFID systems using qubits. Cryptography.

[ref-2] Avoine G, Bingöl MA, Kardaş S, Lauradoux C, Martin B (2011). A framework for analyzing RFID distance bounding protocols. Journal of Computer Security.

[ref-3] Avoine G, Boureanu I, Gérault D, Hancke GP, Lafourcade P, Onete C (2021). From relay attacks to distance-bounding protocols. Security of ubiquitous computing systems.

[ref-4] Avoine G, Bultel X, Gambs S, Gerault D, Lafourcade P, Onete C, Robert J-M (2017). A terrorist-fraud resistant and extractor-free anonymous distance-bounding protocol.

[ref-5] Avoine G, Floerkemeier C, Martin B (2009). RFID distance bounding multistate enhancement.

[ref-6] Avoine G, Lauradoux C, Martin B (2011). How secret-sharing can defeat terrorist fraud.

[ref-7] Avoine G, Tchamkerten A (2009). An efficient distance bounding RFID authentication protocol: balancing false-acceptance rate and memory requirement.

[ref-8] Avoine G, Bingöl MA, Boureanu I, Čapkun S, Hancke G, Kardaş S, Kim CH, Lauradoux C, Martin B, Munilla J (2018). Security of distance-bounding: a survey. ACM Computing Surveys.

[ref-9] Bellare M, Rogaway P (1993). Entity authentication and key distribution.

[ref-10] Bernstein DJ (2005). The Poly1305-AES message-authentication code.

[ref-11] Boureanu I, Mitrokotsa A, Vaudenay S (2013a). Secure and lightweight distance-bounding.

[ref-12] Boureanu I, Mitrokotsa A, Vaudenay S (2013b). Towards secure distance bounding.

[ref-13] Boureanu I, Mitrokotsa A, Vaudenay S (2015). Practical and provably secure distance-bounding. Journal of Computer Security.

[ref-14] Brands S, Chaum D (1993). Distance-bounding protocols.

[ref-15] Brelurut A, Gerault D, Lafourcade P (2015). Survey of distance bounding protocols and threats.

[ref-16] Bussard L (2004). Trust establishment protocols for communicating devices. PhD thesis.

[ref-17] Bussard L, Bagga W (2005). Distance-bounding proof of knowledge to avoid real-time attacks.

[ref-18] Čapkun S, Buttyán L, Hubaux J-P (2003). SECTOR: secure tracking of node encounters in multi-hop wireless networks.

[ref-19] Capkun S, El Defrawy K, Tsudik G (2011). Group distance bounding protocols.

[ref-20] Capkun S, Hubaux J-P (2005). Secure positioning of wireless devices with application to sensor networks.

[ref-21] Capkun S, Hubaux J-P (2006). Secure positioning in wireless networks. IEEE Journal on Selected Areas in Communications.

[ref-22] Chandran N, Goyal V, Moriarty R, Ostrovsky R (2014). Position-based cryptography. SIAM Journal on Computing.

[ref-23] Chiang JT, Haas JJ, Hu Y-C (2009). Secure and precise location verification using distance bounding and simultaneous multilateration.

[ref-24] Cremers C, Rasmussen KB, Schmidt B, Capkun S (2012). Distance hijacking attacks on distance bounding protocols.

[ref-25] Debant A, Delaune S, Wiedling C (2020). So near and yet so far-Symbolic verification of distance-bounding protocols. PhD thesis.

[ref-26] Desmedt Y (1988). Major security problems with the ‘unforgeable’(Feige)-Fiat-Shamir proofs of identity and how to overcome them.

[ref-27] Desmedt Y, Goutier C, Bengio S (1987). Special uses and abuses of the Fiat-Shamir passport protocol.

[ref-28] Drimer S, Murdoch SJ (2007). Keep your enemies close: distance bounding against smartcard relay attacks.

[ref-29] Guillou LC, Quisquater J-J (1988). A practical zero-knowledge protocol fitted to security microprocessor minimizing both transmission and memory.

[ref-30] Guttman JD, Thayer FJ, Zuck LD (2004). The faithfulness of abstract protocol analysis: Message authentication. Journal of Computer Security.

[ref-31] Hancke GP (2012). Distance-bounding for RFID: effectiveness of ‘terrorist fraudin the presence of bit errors.

[ref-32] Hancke GP, Kuhn MG (2005). An RFID distance bounding protocol.

[ref-33] Håstad J, Impagliazzo R, Levin LA, Luby M (1999). A pseudorandom generator from any one-way function. SIAM Journal on Computing.

[ref-34] Hu Y-C, Perrig A, Johnson DB (2003). Packet leashes: a defense against wormhole attacks in wireless networks.

[ref-35] Jannati H, Ardeshir-Larijani E (2016). Detecting relay attacks on RFID communication systems using quantum bits. Quantum Information Processing.

[ref-36] Kardaş S, ÇElik S, YıLdıZ M, Levi A (2012). PUF-enhanced offline RFID security and privacy. Journal of Network and Computer Applications.

[ref-37] Kardaş S, Kiraz MS, Bingöl MA, Demirci H (2011). A novel RFID distance bounding protocol based on physically unclonable functions.

[ref-38] Kılınç H, Vaudenay S (2018). Formal analysis of distance bounding with secure hardware.

[ref-39] Kim CH, Avoine G (2011). RFID distance bounding protocols with mixed challenges. IEEE Transactions on Wireless Communications.

[ref-40] Kim CH, Avoine G, Koeune F, Standaert F-X, Pereira O (2008). The swiss-knife RFID distance bounding protocol.

[ref-41] Kuhn MG (2004). An asymmetric security mechanism for navigation signals.

[ref-42] Kuhn M, Luecken H, Tippenhauer NO (2010). UWB impulse radio based distance bounding.

[ref-43] Lin, Guowei Wu C (2013). Enhancing the attacking efficiency of the node captureattack in WSN: a matrix approach. Journal of Supercomput, Springer Science &Business Media.

[ref-44] Lo N-W, Yeh K-H (2010). De-synchronization attack on RFID authentication protocols.

[ref-45] Meadows C, Poovendran R, Pavlovic D, Chang L, Syverson P (2007). Distance bounding protocols: authentication logic analysis and collusion attacks. Secure localization and time synchronization for wireless sensor and ad hoc networks.

[ref-46] Meadows C, Syverson P, Chang L (2006). Towards more efficient distance bounding protocols for use in sensor networks.

[ref-47] Motara YM, Irwin B (2016). Sha-1 and the strict avalanche criterion.

[ref-48] Munilla J, Ortiz A, Peinado A (2006). Distance bounding protocols with void-challenges for RFID.

[ref-49] Munilla J, Peinado A (2008). Distance bounding protocols for RFID enhanced by using void-challenges and analysis in noisy channels. Wireless Communications and Mobile Computing.

[ref-50] Nikov V, Vauclair M (2008). Yet another secure distance-bounding protocol. SECRYPT.

[ref-51] Peris-Lopez P, Hernandez-Castro JC, Dimitrakakis C, Mitrokotsa A, Tapiador JM (2009). Shedding light on RFID distance bounding protocols and terrorist fraud attacks.

[ref-52] Rasmussen KB, Čapkun S (2008). Location privacy of distance bounding protocols.

[ref-53] Rasmussen KB, Capkun S (2010). Realization of RF Distance Bounding.

[ref-54] Reid J, Nieto JMG, Tang T, Senadji B (2007). Detecting relay attacks with timing-based protocols.

[ref-55] Rowe PD, Guttman JD, Ramsdell JD (2020). Assumption-based analysis of distance-bounding protocols with cpsa. Logic, language, and security.

[ref-56] Sastry N, Shankar U, Wagner D (2003). Secure verification of location claims.

[ref-57] Shmatikov V, Wang M-H (2007). Secure verification of location claims with simultaneous distance modification.

[ref-58] Silberschneider R, Korak T, Hutter M (2013). Access without permission: a practical RFID relay attack.

[ref-59] Singelee D, Preneel B (2005). Location verification using secure distance bounding protocols.

[ref-60] Singelée D, Preneel B (2007). Distance bounding in noisy environments.

[ref-61] Sobti R, Geetha G (2012). Cryptographic hash functions: a review. International Journal of Computer Science Issues.

[ref-62] Strasser M, Danev B, Čapkun S (2010). Detection of reactive jamming in sensor networks. ACM Transactions on Sensor Networks.

[ref-63] Tague P, Poovendran R (2008). Modeling node capture attacks in wireless sensor networks.

[ref-64] Tippenhauer NO, Čapkun S (2009). Id-based secure distance bounding and localization.

[ref-65] Tu Y-J, Piramuthu S (2007). RFID distance bounding protocols.

[ref-66] Tuyls P, Batina L (2006). RFID-tags for anti-counterfeiting.

[ref-67] Waters B, Felten E (2003). Secure, private proofs of location. Department of Computer Science, Princeton University, Tech. Rep. TR-667-03.

[ref-68] Wei G, Zhang H, Wang Y (2016). A new relay attack on distance bounding protocols and its solution with time-stamped authentication for RFID. Wuhan University Journal of Natural Sciences.

