# Supplementary material for: A new secure authentication based distance bounding protocol
Source: PeerJ Comput Sci. 2021 May 6;7:e517. doi: 10.7717/peerj-cs.517 (PMC8114813; doi:10.7717/peerj-cs.517)
Supplement: Supplemental Information 2 [file peerj-cs-07-517-s002.docx]

| **Code** | **Explanation** |
| --- | --- |
| #!/usr/bin/env python3  import hmac, hashlib, random, os, datetime  from bitstring import BitArray  def enc():  bits_size = 512#bits  byte_size = int(bits_size / 8)#bytes  x = os.urandom(byte_size)  m = os.urandom(byte_size)  Np = os.urandom(byte_size)  Nv = os.urandom(byte_size)  hmac_code = hmac.new(key=x, msg=Np, digestmod=hashlib.sha512)  a = hmac_code.digest()  hmac_code = hmac.new(key=m, msg=Nv, digestmod=hashlib.sha512)  b = hmac_code.digest()  ai = os.urandom(byte_size)  betai = ""  for i in range(len(BitArray(ai).bin)):  if BitArray(ai).bin[i] == '0':  betai = betai + BitArray(a).bin[i]  else:  betai = betai + BitArray(b).bin[i]  return betai    def verifier(a,b):  count = 0  for i in range(len(a)):  if a[i] != b[i]:  count = count + 1  return count    def main():  begin_time = datetime.datetime.now()  e = enc()  received_e = "11110111000010000000000000101101011001110000010110100101101000001101010011100001010010100010011101010101010111111000011011100111010111011110010101100001101011011010010110010100100101100110101001001110100101111111000001110011101001101111111110110100111100010010101111100111101110111010001101100011111001011111010010001001101101101001001111010010111011100110011111111001110101110110001111001100111010100001000111000100111000101010010011011110110100011001010101101110101001100000001000111110100110100111110101011000"  print("hour:minute:second:microsecond")  print(datetime.datetime.now() - begin_time)  print()  print(e)    print("Verifying!")  count = verifier(e, received_e)  print("Number of Errors: {}".format(count))  efficiency = count/len(e) * 100  if efficiency>70  print("Efficiency : {}".format(efficiency))  else: print("Protocol Rejected")  return    if __name__ == "__main__":  main() | Pre Computation and Initialization Phase  Bit size define – 512 bits  Bits converted to bytes  Generate random key “x”  Generate of random key “m”  Generate Nonce “Np”  Generate Nonce “Nv”  Encrypt nonce “Np” with “x” to get random number “**a”** (sha512 is used)  Encrypt nonce “Nv” with “m” to get random number “**b”** (sha512 is used)  Generate random number “αi” – 512 bit.  Rapid Bit Exchange Phase Starts  Sending challenge bit “αi”; getting response bit of “a” or “b”.  Values of “a” or “b” stored in “βi”.  Authentication Phase  Verify each received bit of “βi” against pre-computed bits “βi”  Count the bits not matching pre-computed bits of βi.  Return the number of miss-matched bits.  Main Function  Compute time of protocol  Calling of encryption function (PCP, IP and RBEP)  Self-given value of received βi (from prover side)  Time of protocol  Print output bit string of “βi”  Print Verifying  Call validation function (AP)  Print number of miss-matched bits.  Determine percentage error.  Check percentage error against a given threshold.  Accept or reject Protocol  End |
